# Supplementary material for: The Evolution and Characterization of the RNA Interference Pathways in Lophotrochozoa
Source: Genome Biol Evol. 2024 May 7;16(5):evae098. doi: 10.1093/gbe/evae098 (PMC11114477; doi:10.1093/gbe/evae098)
Supplement: evae098_Supplementary_Data [file evae098_supplementary_data.zip › Supplementary_materials.zip!/selected_miRNAs/NoGe_selected_miRNAs/mirdeep_pdf_files/NMRB01000654.1_Notospermus_geniculatus_strain_Ushimado_scaffold654,_whole_genome_shotgun_sequence_17940.pdf]

Provisional ID	:	NMRB01000654.1_Notospermus_geniculatus_strain_Ushimado_scaffold654,_whole_genome_shotgun_sequence_17940
Score total	:	29978.2
Score for star read(s)	:	3.9
Score for read counts	:	29969.7
Score for mfe	:	1.6
Score for randfold	:	0
Score for cons. seed	:	3
Total read count	:	58796
Mature read count	:	57058
Loop read count	:	0
Star read count	:	1738

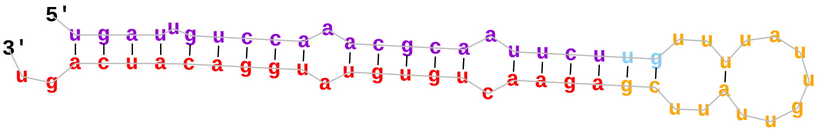

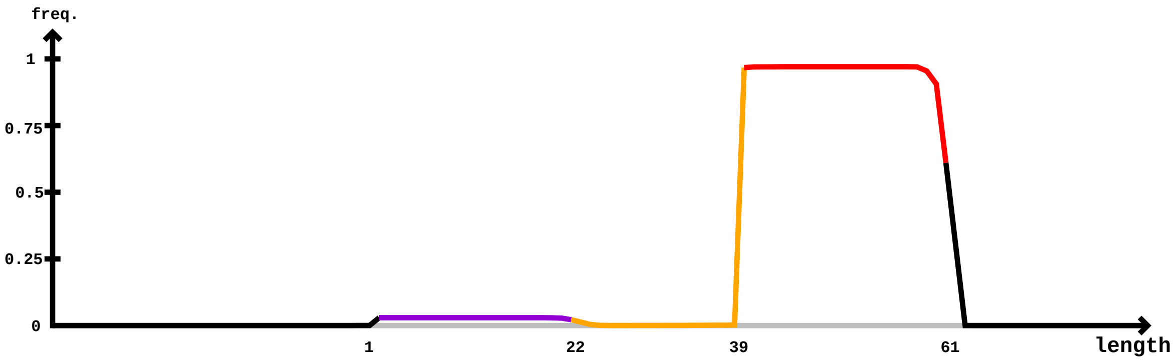

Star **Mature**

```
5'- gcaucuuccuucguaagaaagguuacaggcggugauuguccaaacgcaauucuuguuuuauuguuauucgagaacuguguauggacaucagugcuugccggccgguuucgac  -3'   obs
    gcaucuuccuucguaagaaagguuacaggcggugauuguccaaacgcaauucuuguuuuauuguuauucgagaacuguguauggacaucagugcuugccggccgguuucgac         exp
    ..........(((..(((..((((.((((((.((((.(((((.(((((.((((((..(.......)..)))))).))))).))))))))).))))))..))))..)))))).   reads   mm   sample
    ..............................ggugauuguccaaacgcaauuc............................................................   1       0    Fm3
    ................................ugauuguccaaacAcaau..............................................................   1       1    Fm3
    ................................ugauuguccaaacgcaaA..............................................................   1       1    Fm3
    ................................ugauuguccaaacgcaau..............................................................   13      0    Fm3
    ................................ugauuguccUaacgcaau..............................................................   1       1    Fm3
    ................................ugauuguccaaacgcaauu.............................................................   50      0    Fm3
    ................................ugauuguccaaacgcaaCu.............................................................   1       1    Fm3
    ................................ugauuguccaCacgcaauu.............................................................   1       1    Fm3
    ................................ugauuguccaaacgcaauA.............................................................   10      1    Fm3
    ................................ugauuguccaaacgcaaAuc............................................................   4       1    Fm3
    ................................ugauuguccaaacgcaUuuc............................................................   2       1    Fm3
    ................................ugauuguccaaacgcaauuc............................................................   181     0    Fm3
    ................................ugauuguccaaacgcaaCuc............................................................   3       1    Fm3
    ................................ugauuguccaaacgcaaGuc............................................................   1       1    Fm3
    ................................ugaAuguccaaacgcaauuc............................................................   1       1    Fm3
    ................................Agauuguccaaacgcaauuc............................................................   1       1    Fm3
    ................................ugauuguccaaacgcaaCucu...........................................................   1       1    Fm3
    ................................ugauuguccaaacgcaaGucu...........................................................   3       1    Fm3
    ................................ugauuguccaaacgcaauucu...........................................................   124     0    Fm3
    ................................ugauuguccGaacgcaauucu...........................................................   1       1    Fm3
    ................................ugauuguccaUacgcaauucu...........................................................   1       1    Fm3
    ................................ugauuguccaaacgcaauucuu..........................................................   6       0    Fm3
    ................................ugauuguccGaacgcaauucuug.........................................................   1       1    Fm3
    ................................uNauuguccaaacgcaauucuug.........................................................   1       1    Fm3
    ................................ugauuguccaaacgcaauucuug.........................................................   43      0    Fm3
    ................................ugauuguccaaacgcaauucuugu........................................................   2       0    Fm3
    .................................gauuguccaaacgcaauucuug.........................................................   1       0    Fm3
    .............................................................uguuauucgagaacuguguauggacaucagu....................   1       0    Fm3
    ................................................................uauucgagaacuguguauggacaucagu....................   3       0    Fm3
    .................................................................auucgagaacuguguauggacauca......................   2       0    Fm3
    ..................................................................uucgagaacuguguauggacauc.......................   3       0    Fm3
    ...................................................................ucgagaacuguguauggacau........................   8       0    Fm3
    ...................................................................ucgagaacuguguauggacauU.......................   7       1    Fm3
```

```
                                Star                                      Mature
gcaucuuccuucguaagaaagguuacaggcggugauuguccaaacgcaauucuuguuuuauuguuauucgagaacuguguauggacaucagugcuugccggccgguuucgac
...................................................................ucgagaacuguguauggacauc.......................    8      0    Fm3
...................................................................ucgagaacuguguauggacauca......................    3      0    Fm3
.....................................................................Cagaacuguguauggacauc.......................    2      1    Fm3
.....................................................................Cagaacuguguauggacaucag.....................    3      1    Fm3
.....................................................................Cagaacuguguauggacaucagu....................    10     1    Fm3
......................................................................agaacuguguauggacau........................    20     0    Fm3
......................................................................agaacuguguauggacauc.......................    494    0    Fm3
......................................................................agaacuguguauggacUuc.......................    1      1    Fm3
......................................................................agaacuguguauggacCuc.......................    1      1    Fm3
......................................................................agaacuguguauggacauA.......................    37     1    Fm3
......................................................................agaacuguguauggacaAc.......................    2      1    Fm3
......................................................................agaacuguguauggacauU.......................    23     1    Fm3
......................................................................agaacuguguauggacaCc.......................    2      1    Fm3
......................................................................agaacuguUuauggacauc.......................    1      1    Fm3
......................................................................agaacuguguCuggacauc.......................    2      1    Fm3
......................................................................agaacNguguauggacauca......................    1      1    Fm3
......................................................................agaacuAuguauggacauca......................    1      1    Fm3
......................................................................agaacuguguauggacaAca......................    5      1    Fm3
......................................................................agaacuguguaCggacauca......................    1      1    Fm3
......................................................................agaacuguguauggacauUa......................    1      1    Fm3
......................................................................agaacuguguaGggacauca......................    1      1    Fm3
......................................................................agaacugCguauggacauca......................    1      1    Fm3
......................................................................agaacuguguauggacGuca......................    5      1    Fm3
......................................................................agaacuguguauggacauca......................    2144   0    Fm3
......................................................................agaacuguguUuggacauca......................    1      1    Fm3
......................................................................agaacugAguauggacauca......................    1      1    Fm3
......................................................................agaacuguguauggacaGca......................    7      1    Fm3
......................................................................agaacuguguauggacUuca......................    3      1    Fm3
......................................................................agaacuguguauggacaCca......................    17     1    Fm3
......................................................................agaacuguguauggacaucC......................    1      1    Fm3
......................................................................agaacuguguauggacaucU......................    21     1    Fm3
......................................................................Ngaacuguguauggacauca......................    1      1    Fm3
......................................................................agaacuguguGuggacauca......................    2      1    Fm3
......................................................................Ugaacuguguauggacauca......................    2      1    Fm3
......................................................................agaacuguguauggacCuca......................    5      1    Fm3
......................................................................agaacuguguauUgacauca......................    2      1    Fm3
......................................................................agaacuguguauggCcaucag.....................    2      1    Fm3
......................................................................agaacugGguauggacaucag.....................    1      1    Fm3
......................................................................agaacuguguauUgacaucag.....................    3      1    Fm3
......................................................................agaacuguguUuggacaucag.....................    12     1    Fm3
......................................................................agaGcuguguauggacaucag.....................    2      1    Fm3
......................................................................agUacuguguauggacaucag.....................    2      1    Fm3
......................................................................agaacuguguauggacUucag.....................    24     1    Fm3
......................................................................agaacuguguCuggacaucag.....................    20     1    Fm3
......................................................................agaCcuguguauggacaucag.....................    1      1    Fm3
......................................................................agaacugugCauggacaucag.....................    10     1    Fm3
......................................................................agaaGuguguauggacaucag.....................    2      1    Fm3
......................................................................agaacuguguauAgacaucag.....................    5      1    Fm3
......................................................................agaacNguguauggacaucag.....................    2      1    Fm3
......................................................................agaacuguguauggacGucag.....................    12     1    Fm3
......................................................................Ugaacuguguauggacaucag.....................    13     1    Fm3
......................................................................agaacugugGauggacaucag.....................    1      1    Fm3
......................................................................agaacuguUuauggacaucag.....................    2      1    Fm3
......................................................................agaacuguguaugCacaucag.....................    1      1    Fm3
......................................................................agaacuguguauggacaucGg.....................    3      1    Fm3
......................................................................agGacuguguauggacaucag.....................    4      1    Fm3
......................................................................agaUcuguguauggacaucag.....................    1      1    Fm3
......................................................................agaacuguguaAggacaucag.....................    1      1    Fm3
......................................................................agaacuguguauggacauGag.....................    2      1    Fm3
......................................................................agaacuguguauggacaGcag.....................    75     1    Fm3
......................................................................agaacugCguauggacaucag.....................    2      1    Fm3
......................................................................agaacuguAuauggacaucag.....................    1      1    Fm3
......................................................................agaacuguguauggaAaucag.....................    2      1    Fm3
......................................................................agaacuguguGuggacaucag.....................    26     1    Fm3
......................................................................agaacuguguauggUcaucag.....................    1      1    Fm3
......................................................................agaacuguguauggaGaucag.....................    3      1    Fm3
......................................................................agaacuguguaugUacaucag.....................    3      1    Fm3
......................................................................agaacuguguauggaUaucag.....................    5      1    Fm3
......................................................................agaacuUuguauggacaucag.....................    2      1    Fm3
......................................................................agaacuguguauggacaucag.....................    13868  0    Fm3
```

Star Mature

gcaucuuccuucguaagaaagguuacaggcggugauuguccaaacgcaauucuuguuuuauuguuauucgagaacuguguauggacaucagugcuugccggccgguuucgac			
....Ggaacuguguauggacaucag.....................	2	1	Fm3
....agaacuguCuauggacaucag.....................	1	1	Fm3
....agaacuguguauggacCucag.....................	22	1	Fm3
....aNaacuguguauggacaucag.....................	1	1	Fm3
....agaacuguguauggGcaucag.....................	5	1	Fm3
....aUaacuguguauggacaucag.....................	1	1	Fm3
....agaacCguguauggacaucag.....................	1	1	Fm3
....agaacuguguauggacauUag.....................	6	1	Fm3
....Ngaacuguguauggacaucag.....................	4	1	Fm3
....agaacuguguauggacaucUg.....................	2	1	Fm3
....agaacugugAauggacaucag.....................	1	1	Fm3
....aAaacuguguauggacaucag.....................	6	1	Fm3
....agaacuguguauggacaucaU.....................	38	1	Fm3
....agaaAuguguauggacaucag.....................	2	1	Fm3
....agaacuguguauggacaucCg.....................	2	1	Fm3
....agaacuguguaugAacaucag.....................	5	1	Fm3
....agaacuguguauggacaCcag.....................	84	1	Fm3
....agaacuNuguauggacaucag.....................	1	1	Fm3
....agaaUuguguauggacaucag.....................	1	1	Fm3
....Cgaacuguguauggacaucag.....................	2	1	Fm3
....agaacuguguauggacaAcag.....................	58	1	Fm3
....agaacuguguauggacauAagu....................	5	1	Fm3
....agaacuguguUuggacaucagu....................	20	1	Fm3
....Cgaacuguguauggacaucagu....................	1	1	Fm3
....aUaacuguguauggacaucagu....................	4	1	Fm3
....agaacNguguauggacaucagu....................	4	1	Fm3
....agaacuguguauggaUaucagu....................	2	1	Fm3
....agaGcuguguauggacaucagu....................	2	1	Fm3
....agaacuguguauUgacaucagu....................	3	1	Fm3
....agaCcuguguauggacaucagu....................	2	1	Fm3
....agaacuguguaugAacaucagu....................	3	1	Fm3
....agaacuguguauggacaAcagu....................	81	1	Fm3
....agaacuguguauggacGucagu....................	23	1	Fm3
....agaacuguguauggacaucaUu....................	13	1	Fm3
....agaacuguguauggacaucCgu....................	2	1	Fm3
....agaacuguguGuggacaucagu....................	22	1	Fm3
....agaacuguguauggGcaucagu....................	5	1	Fm3
....agaacuAuguauggacaucagu....................	3	1	Fm3
....agaacuNuguauggacaucagu....................	2	1	Fm3
....agaacuguguCuggacaucagu....................	19	1	Fm3
....agaacuguguaCggacaucagu....................	4	1	Fm3
....agaacuguguauggCcaucagu....................	2	1	Fm3
....agUacuguguauggacaucagu....................	1	1	Fm3
....agaacuguCuauggacaucagu....................	3	1	Fm3
....agaacuguguauggacaGcagu....................	86	1	Fm3
....Ugaacuguguauggacaucagu....................	17	1	Fm3
....agaacugugGauggacaucagu....................	1	1	Fm3
....agaaAuguguauggacaucagu....................	1	1	Fm3
....agaUcuguguauggacaucagu....................	1	1	Fm3
....Ggaacuguguauggacaucagu....................	3	1	Fm3
....agaacuguguauggaGaucagu....................	1	1	Fm3
....agaacGguguauggacaucagu....................	1	1	Fm3
....agaacuguguauggacauGagu....................	1	1	Fm3
....agaaUuguguauggacaucagu....................	5	1	Fm3
....agaacuguguauggacaCcagu....................	95	1	Fm3
....agaacuguguauggacaucaAu....................	4	1	Fm3
....agaacCguguauggacaucagu....................	1	1	Fm3
....agaacuguUuauggacaucagu....................	1	1	Fm3
....agaacuguguauggacaucagu....................	18758	0	Fm3
....agaacuguguauCgacaucagu....................	5	1	Fm3
....agaacuguguauggUcaucagu....................	4	1	Fm3
....agaacuguguauggacUucagu....................	26	1	Fm3
....agaacuguguauggacaucagA....................	34	1	Fm3
....agaacuguguauggacaucaCu....................	1	1	Fm3
....agaacuguAuauggacaucagu....................	4	1	Fm3
....aAaacuguguauggacaucagu....................	8	1	Fm3
....agaacuUuguauggacaucagu....................	1	1	Fm3
....agaacugCguauggacaucagu....................	4	1	Fm3
....agaacAguguauggacaucagu....................	3	1	Fm3
....Ngaacuguguauggacaucagu....................	6	1	Fm3

```
                                Star                                        Mature
gcaucuuccuucguaagaaagguuacaggcggugauuguccaaacgcaauucuuguuuuauuguuauucgagaacuguguauggacaucagugcuugccggccgguuucgac
......................................................................agaacuguguauAgacaucagu....................    2     1   Fm3
......................................................................agaacuguguaAggacaucagu....................    2     1   Fm3
......................................................................agGacuguguauggacaucagu....................    8     1   Fm3
......................................................................agaacuguguauggacaucGgu....................    2     1   Fm3
......................................................................agaacuguguauggacaucagC....................    1     1   Fm3
......................................................................agaacugugAauggacaucagu....................    1     1   Fm3
......................................................................agaacuguguauggacCucagu....................    32    1   Fm3
......................................................................agaacuguguauggacauUagu....................    11    1   Fm3
......................................................................agaacuguguauggaAaucagu....................    1     1   Fm3
......................................................................agaacuguguaugCacaucagu....................    5     1   Fm3
......................................................................agaacugAguauggacaucagu....................    2     1   Fm3
......................................................................agaacuguguaugUacaucagu....................    9     1   Fm3
......................................................................agaacuguguauggacaucaguU...................    112   1   Fm3
......................................................................agaacuguguauggacaucagug...................    1     0   Fm3
......................................................................agaacuguguauggacaucaguA...................    15    1   Fm3
.......................................................................gaacuguguauggacauc.......................    3     0   Fm3
.......................................................................gaacugugCauggacauc.......................    1     1   Fm3
.......................................................................gaacuguguauggacauca......................    1     0   Fm3
.......................................................................gaacuguguaugUacaucag.....................    1     1   Fm3
.......................................................................gaacuguguaAggacaucag.....................    1     1   Fm3
.......................................................................gaacuguguauggacaucag.....................    17    0   Fm3
.......................................................................gaacuguguauggacGucagu....................    1     1   Fm3
.......................................................................gaacuguguauggacaucagu....................    83    0   Fm3
.........................................................................acuguguauggacaucagug...................    6     0   Fm3
..........................................................................cuguguauggacaucagA....................    1     1   Fm3
..........................................................................cuguguauggacaucagu....................    16    0   Fm3

...............................Cugauuguccaaacgcaauucu...........................................................    1     1   Mm3
................................ugauuguccaaacgcaau..............................................................    1     0   Mm3
................................ugauuguccaaacgcaauuc............................................................    24    0   Mm3
................................ugauuguccaaacUcaauucu...........................................................    1     1   Mm3
................................ugauuguccaaacgcaauucu...........................................................    57    0   Mm3
................................ugauuguccaaacgcaauucuu..........................................................    2     0   Mm3
................................ugauuguccaaacgcaauucuug.........................................................    19    0   Mm3
..............................................................guuauucgagaacuguguauggacaucagu....................    1     0   Mm3
...............................................................uuauucgagaacuguguauggacauc........................    1     0   Mm3
.................................................................auucgagaacuguguauggacaucag......................    2     0   Mm3
.................................................................auucgagaacuguguauggacaucagu.....................    1     0   Mm3
....................................................................gagaacuguguauggacaucag......................    1     0   Mm3
.....................................................................Cagaacuguguauggacaucagu....................    5     1   Mm3
......................................................................agaacuguguauggacau........................    1     0   Mm3
......................................................................agaacuguguauggacauc.......................    95    0   Mm3
......................................................................agaacuguguauggacauA.......................    2     1   Mm3
......................................................................agaacuguguauggacGuc.......................    1     1   Mm3
......................................................................agaacuguguauggacauU.......................    8     1   Mm3
......................................................................agaacuguguauggacaGca......................    1     1   Mm3
......................................................................agaacuguguauggacauca......................    195   0   Mm3
......................................................................agaacuguguauggacCuca......................    1     1   Mm3
......................................................................agaacuguguauggacaucG......................    1     1   Mm3
......................................................................agaUcuguguauggacauca......................    1     1   Mm3
......................................................................agaacuguguauggacaucU......................    7     1   Mm3
......................................................................agaacuguguauggacaAca......................    1     1   Mm3
......................................................................agaacuguguauggacauUag.....................    2     1   Mm3
......................................................................agaacuguguauggacaucaA.....................    1     1   Mm3
......................................................................agaacugAguauggacaucag.....................    1     1   Mm3
......................................................................agaacNguguauggacaucag.....................    1     1   Mm3
......................................................................agaacuguguauggacaAcag.....................    4     1   Mm3
......................................................................agaacuguguauggacaGcag.....................    4     1   Mm3
......................................................................agaacuAuguauggacaucag.....................    1     1   Mm3
......................................................................agaacuguguCuggacaucag.....................    1     1   Mm3
......................................................................agaacuguguauggacaucaU.....................    10    1   Mm3
......................................................................agaacuguguauggacCucag.....................    2     1   Mm3
......................................................................Ugaacuguguauggacaucag.....................    2     1   Mm3
......................................................................agaUcuguguauggacaucag.....................    1     1   Mm3
......................................................................agaacuguguauggacaCcag.....................    9     1   Mm3
......................................................................agaacuguguauggacGucag.....................    2     1   Mm3
......................................................................agaacuguguauggacaucaC.....................    1     1   Mm3
......................................................................agaacuguguauCgacaucag.....................    1     1   Mm3
......................................................................agaacuguguUuggacaucag.....................    1     1   Mm3
......................................................................agaacuguguauggacaucag.....................    1085  0   Mm3
```

Star **Mature**

gcaucuuccuucguaagaaagguuacaggcggugauuguccaaacgcaauucuuguuuuauuguuauucgagaacuguguauggacaucagugcuugccggccgguuucgac			
....agaacCguguauggacaucag.....................	1	1	Mm3
....agaacuguguauggacaucGg.....................	2	1	Mm3
....agaacuguguauggacaucUg.....................	1	1	Mm3
....agaacuguguauggacUucag.....................	5	1	Mm3
....agaacugugAauggacaucagu....................	1	1	Mm3
....agaacuguguauggacauAagu....................	1	1	Mm3
....Ugaacuguguauggacaucagu....................	6	1	Mm3
....agaUcuguguauggacaucagu....................	1	1	Mm3
....aAaacuguguauggacaucagu....................	1	1	Mm3
....agaacuguguUuggacaucagu....................	7	1	Mm3
....agaacugCguauggacaucagu....................	1	1	Mm3
....agNacuguguauggacaucagu....................	1	1	Mm3
....agaacuNuguauggacaucagu....................	1	1	Mm3
....agaaUuguguauggacaucagu....................	1	1	Mm3
....agaacuguguauggacUucagu....................	6	1	Mm3
....agCacuguguauggacaucagu....................	2	1	Mm3
....agaacuguguauggacaucagG....................	9	1	Mm3
....agaacuguUuauggacaucagu....................	3	1	Mm3
....agaacAguguauggacaucagu....................	1	1	Mm3
....agaacuguguauggacauUagu....................	5	1	Mm3
....agaacuUuguauggacaucagu....................	1	1	Mm3
....aCaacuguguauggacaucagu....................	1	1	Mm3
....agaacuguguauggacGucagu....................	5	1	Mm3
....agaacCguguauggacaucagu....................	1	1	Mm3
....agaacuguguauggaUaucagu....................	2	1	Mm3
....agaacuCuguauggacaucagu....................	2	1	Mm3
....agaacuguguGuggacaucagu....................	9	1	Mm3
....agaacugGguauggacaucagu....................	1	1	Mm3
....agaacuguguauggacCucagu....................	7	1	Mm3
....agaacuguguauggacaCcagu....................	30	1	Mm3
....aUaacuguguauggacaucagu....................	1	1	Mm3
....agaacuguguauggacaucagu....................	5823	0	Mm3
....agaacuguguauggacaGcagu....................	20	1	Mm3
....agGacuguguauggacaucagu....................	5	1	Mm3
....Ggaacuguguauggacaucagu....................	2	1	Mm3
....agaacuguguauggGcaucagu....................	2	1	Mm3
....agaacuguguauggacaAcagu....................	27	1	Mm3
....agaaGuguguauggacaucagu....................	2	1	Mm3
....agUacuguguauggacaucagu....................	1	1	Mm3
....agaacuguguaAggacaucagu....................	1	1	Mm3
....Ngaacuguguauggacaucagu....................	4	1	Mm3
....agaacuguguauggacaucaUu....................	5	1	Mm3
....agaacuAuguauggacaucagu....................	1	1	Mm3
....agaacuguguauggacaucagC....................	6	1	Mm3
....agaacuguguaugAacaucagu....................	1	1	Mm3
....agaacugugCauggacaucagu....................	2	1	Mm3
....agaacuguguCuggacaucagu....................	5	1	Mm3
....agaacuguguauggacaucagA....................	12	1	Mm3
....agaacuguguauggacaucaguA...................	6	1	Mm3
....agaacuguguauggacaucaguU...................	24	1	Mm3
....agaacuguguauggacaucagug...................	1	0	Mm3
....agaacuguguauggacaucagugU..................	2	1	Mm3
....gaacuguguauggacaucag.....................	1	0	Mm3
....gaacuguguauggacaucaUu....................	1	1	Mm3
....gaacuguguauggacaucagu....................	22	0	Mm3
....gaacuguguauggacauAagu....................	1	1	Mm3
....aacuguguauggacaucagu....................	1	0	Mm3
....cuguguauggacaucagu....................	2	0	Mm3
..............................ggugauuguccaaacgcaauuc..	10	0	Fm2
................................ugauuguccaaacgcaauuc..	15	0	Fm2
................................ugauuguccaaacgcaCuuc..	1	1	Fm2
................................ugauuguccaaacgcaaGucu...	1	1	Fm2
................................ugauuguccaaacgcaauucG...	2	1	Fm2
................................ugauuguccaUacgcaauucu...	1	1	Fm2
................................ugauuguccaaacgcaauucu...	203	0	Fm2
................................ugauuguccaaacgcaaCucu...	2	1	Fm2
................................ugauuguccaaacgcaauucuu..	11	0	Fm2
................................ugauuguccaaacgcaaAucuu..	1	1	Fm2
................................ugauuguccaaacgcaauucuug...	27	0	Fm2

Star Mature

```
gcaucuuccuucguaagaaagguuacaggcggugauuguccaaacgcaauucuuguuuuauuguuauucgagaacuguguauggacaucagugcuugccggccgguuucgac
................................ugauuguccaaacgcaauucuugu........................................................    11    0    Fm2
.......................................................uuuuauuguuauucgagaacuguguauggGcaucagu....................    1     1    Fm2
.......................................................uuuuauuguuauucgagaacuguguauggacaucagu....................    8     0    Fm2
...........................................................auuguuauucgagaacuguguauggacauGagu....................    1     1    Fm2
...........................................................auuguuauucgagaacuguguauggacaucagu....................    10    0    Fm2
...........................................................auuguuauucgagaacGguguauggacaucagu....................    1     1    Fm2
......................................................................agaacuguguauggacauc.......................    13    0    Fm2
......................................................................agaacuguguauggacauca......................    17    0    Fm2
......................................................................agaacuguguauggacaGca......................    1     1    Fm2
......................................................................agaacuguguauggacaAca......................    1     1    Fm2
......................................................................agaacuguguauggacCuca......................    1     1    Fm2
......................................................................agaacuguguauggacaucag.....................    158   0    Fm2
......................................................................agaacuguguauggacaCcag.....................    2     1    Fm2
......................................................................agaacuguguauggacaGcag.....................    2     1    Fm2
......................................................................agaacuguguCuggacaucagu....................    3     1    Fm2
......................................................................agaacuguguauggacaucagu....................    952   0    Fm2
......................................................................agaacuguguauggacUucagu....................    2     1    Fm2
......................................................................agaacuguguauggacauUagu....................    1     1    Fm2
......................................................................Ngaacuguguauggacaucagu....................    1     1    Fm2
......................................................................agaacuguguauggacaAcagu....................    8     1    Fm2
......................................................................agaacuguguauggacCucagu....................    1     1    Fm2
......................................................................Ugaacuguguauggacaucagu....................    2     1    Fm2
......................................................................agaaUuguguauggacaucagu....................    1     1    Fm2
......................................................................agaacuguguauggacaCcagu....................    5     1    Fm2
......................................................................agaacuguguauggacaGcagu....................    9     1    Fm2
......................................................................agaacuguguauggacGucagu....................    4     1    Fm2
......................................................................agaacuguguauggaUaucagu....................    2     1    Fm2
......................................................................agaacuguguUuggacaucagu....................    1     1    Fm2
......................................................................agaacuguguauggacaucagG....................    5     1    Fm2
......................................................................agaacuguguauggacaucaguU...................    32    1    Fm2
.......................................................................gaacuguguauggacaucagu....................    3     0    Fm2
.......................................................................gaacugugCauggacaucagu....................    1     1    Fm2

...............................gugauuguccaaacgcaauucuu..........................................................    1     0    Fm1
...............................gugauuguccaaacgcaauucuuU.........................................................    1     1    Fm1
................................ugauuguccaaacgcaUu..............................................................    1     1    Fm1
................................ugauuguccaaacgcaau..............................................................    3     0    Fm1
................................ugauuguccaaacgcaauu.............................................................    10    0    Fm1
................................ugauuguccaaacgcaaCuc............................................................    1     1    Fm1
................................ugauuguccaaacgcaaAuc............................................................    1     1    Fm1
................................ugauuguccaaacgcaauuc............................................................    48    0    Fm1
................................ugauuguccaaacgcaauucG...........................................................    2     1    Fm1
................................Ngauuguccaaacgcaauucu...........................................................    2     1    Fm1
................................uAauuguccaaacgcaauucu...........................................................    1     1    Fm1
................................ugauuguccaaacgcaaCucu...........................................................    2     1    Fm1
................................ugauuguccaaacgUaauucu...........................................................    1     1    Fm1
................................ugauuguccaUacgcaauucu...........................................................    2     1    Fm1
................................ugauuguccaaacgcaaGucu...........................................................    4     1    Fm1
................................ugauuguccaaacgcaaAucu...........................................................    4     1    Fm1
................................ugauuguccaaacgcaauucu...........................................................    402   0    Fm1
................................ugauuguccaaacgcaauucuu..........................................................    56    0    Fm1
................................ugauuguccaaacgcaauucuA..........................................................    3     1    Fm1
................................ugauuguccaaacgcaCuucuu..........................................................    2     1    Fm1
................................ugauuguccaaacgcaaCucuu..........................................................    1     1    Fm1
................................ugauuguccaaacgcaaGucuug.........................................................    1     1    Fm1
................................ugauuguccaaacgcaauucuug.........................................................    82    0    Fm1
................................ugauuguccaaacgcaGuucuug.........................................................    1     1    Fm1
................................ugauuguccaaacgcaaAucuugu........................................................    1     1    Fm1
................................ugauuguccaaacgcaauucuugu........................................................    7     0    Fm1
.................................gauuguccaaacgcaauucuug.........................................................    2     0    Fm1
.......................................................uuuuauuguuauucgagaacuguguauggacaucagu....................    1     0    Fm1
..........................................................uauuguuauucgagaacuguguauggacauca......................    1     0    Fm1
..............................................................guuauucgagaacuguguauggacaucag.....................    1     0    Fm1
.................................................................auucgagaacuguguauggaca.........................    2     0    Fm1
.................................................................auucgagaacuguguauggacauc.......................    2     0    Fm1
.................................................................auucgagaacuguguauggacaucag.....................    1     0    Fm1
.................................................................auucgaAaacuguguauggacaucag.....................    1     1    Fm1
.................................................................auucgagaacuguguauggacaucagu....................    9     0    Fm1
.................................................................auucgagaacuguguauggacaucagugcuugccggccg........    1     0    Fm1
.....................................................................Cagaacuguguauggacaucagu....................    1     1    Fm1
```

Star **Mature**

gcaucuuccuucguaagaaagguuacaggcggugauuguccaaacgcaauucuuguuuuauuguuauucgagaacuguguauggacaucagugcuugccggccgguuucgac			
....agaacuguguauggacaGc.......................	1	1	Fm1
....agaacuguguauggacaCc.......................	1	1	Fm1
....agaacuguguauggacauc.......................	86	0	Fm1
....agaacuguguauggacauU.......................	3	1	Fm1
....agaacuguguauggacauca......................	88	0	Fm1
....agGacuguguauggacauca......................	1	1	Fm1
....Ggaacuguguauggacauca......................	1	1	Fm1
....agaacuguguauggacaucG......................	1	1	Fm1
....Ugaacuguguauggacaucag.....................	2	1	Fm1
....agaacuguguauggacaGcag.....................	3	1	Fm1
....agaacuguguauggacCucag.....................	2	1	Fm1
....agaacuguguauggacaucag.....................	507	0	Fm1
....agaacuguguGuggacaucag.....................	1	1	Fm1
....agaacuguguauggacaAcag.....................	2	1	Fm1
....agaacuguguauggacaucaU.....................	9	1	Fm1
....agaacuguguauggacGucag.....................	1	1	Fm1
....agaacuguguaugAacaucag.....................	1	1	Fm1
....agaacuguguauggacUucag.....................	1	1	Fm1
....agaGcuguguauggacaucag.....................	1	1	Fm1
....agaacuguguCuggacaucag.....................	1	1	Fm1
....agaacuguguauggacaCcag.....................	2	1	Fm1
....agaacugugCauggacaucagu....................	1	1	Fm1
....agaacuguguauggacUucagu....................	5	1	Fm1
....agaacuguguauggacGucagu....................	5	1	Fm1
....aAaacuguguauggacaucagu....................	1	1	Fm1
....agaacuguguauggacaucagC....................	1	1	Fm1
....agaacuguguaugAacaucagu....................	1	1	Fm1
....agaacugCguauggacaucagu....................	1	1	Fm1
....Ugaacuguguauggacaucagu....................	1	1	Fm1
....agaacuguguauggacaucagu....................	3568	0	Fm1
....Ngaacuguguauggacaucagu....................	1	1	Fm1
....agaacuguguauggacaucGgu....................	1	1	Fm1
....agaacuguguaCggacaucagu....................	1	1	Fm1
....agaacuguguauggacCucagu....................	3	1	Fm1
....agaacuguguCuggacaucagu....................	5	1	Fm1
....agaacuguguauggacaCcagu....................	24	1	Fm1
....agaacuguguUuggacaucagu....................	3	1	Fm1
....agaacuguguauggCcaucagu....................	1	1	Fm1
....agaacuguguauggacaGcagu....................	23	1	Fm1
....agGacuguguauggacaucagu....................	3	1	Fm1
....Ggaacuguguauggacaucagu....................	1	1	Fm1
....agaacuguguauggacaAcagu....................	15	1	Fm1
....agaacuAuguauggacaucagu....................	3	1	Fm1
....agaacuguguGuggacaucagu....................	4	1	Fm1
....agaGcuguguauggacaucagu....................	1	1	Fm1
....agaacuguguauggacaucUgu....................	1	1	Fm1
....agaacuguguauggacaucagA....................	3	1	Fm1
....agaacuguguauggaUaucagu....................	1	1	Fm1
....agaacuguguauggacauUagu....................	8	1	Fm1
....agUacuguguauggacaucagu....................	1	1	Fm1
....agaacuguguauggacaucagG....................	21	1	Fm1
....agaacugGguauggacaucagu....................	1	1	Fm1
....agaacuguguauggacaucaguA...................	4	1	Fm1
....agaacuguguauggacaucagAg...................	5	1	Fm1
....agaacuguguauggacaucaguU...................	18	1	Fm1
....gaacuguguauggacaucagu....................	12	0	Fm1
................................ugauuguccaaaUgcaauuc..	1	1	Mm1
................................ugauuguccaaacgcaauuc..	33	0	Mm1
................................ugauuguccaaacgcaauucu...	61	0	Mm1
................................ugauuguccaaacgcaaCucu...	1	1	Mm1
................................ugauuguccaaacgcaauucuu..	2	0	Mm1
................................ugauuguccaaacgcaauucuU..	1	1	Mm1
................................ugauuguccaaacgcaauucuug...	19	0	Mm1
................................ugauuguccaaacgcaaCucuug...	1	1	Mm1
................................ugauuguccaaacgcaauucuugu..	7	0	Mm1
....agaacuguguauggacau........................	2	0	Mm1
....agaacuguguauggacauU.......................	4	1	Mm1
....agaacuguguauggacauc.......................	27	0	Mm1
....Ugaacuguguauggacauc.......................	1	1	Mm1

```
                                Star                                    Mature
gcaucuuccuucguaagaaagguuacaggcggugauuguccaaacgcaauucuuguuuuauuguuauucgagaacuguguauggacaucagugcuugccggccgguuucgac
......................................................................agaacuguguauggacauA.......................   6     1   Mm1
......................................................................agaacuguguGuggacauca......................   1     1   Mm1
......................................................................agaacuguguauggacauca......................   122   0   Mm1
......................................................................agaacuguguauggacCuca......................   1     1   Mm1
......................................................................agaacuguguauggacaGcag.....................   1     1   Mm1
......................................................................agaacuguguGuggacaucag.....................   1     1   Mm1
......................................................................agaacuguguauggacaucaA.....................   1     1   Mm1
......................................................................agaacuguguauggacaCcag.....................   2     1   Mm1
......................................................................agaacuguguauggacaucag.....................   266   0   Mm1
......................................................................Ugaacuguguauggacaucag.....................   1     1   Mm1
......................................................................agaacuguguauggacaAcag.....................   1     1   Mm1
......................................................................agaacuguguauggacaucaUu....................   9     1   Mm1
......................................................................agUacuguguauggacaucagu....................   2     1   Mm1
......................................................................agaacuguguCuggacaucagu....................   3     1   Mm1
......................................................................agaacuguguauggacUucagu....................   7     1   Mm1
......................................................................agaacuguguauggaUaucagu....................   1     1   Mm1
......................................................................agaUcuguguauggacaucagu....................   1     1   Mm1
......................................................................agaacuguguauggacaucagA....................   12    1   Mm1
......................................................................agaacuguguauggacGucagu....................   1     1   Mm1
......................................................................agaacuguguauggacCucagu....................   6     1   Mm1
......................................................................agaacuguguauggacaGcagu....................   7     1   Mm1
......................................................................Ugaacuguguauggacaucagu....................   3     1   Mm1
......................................................................agaacuguguauggacaucagG....................   2     1   Mm1
......................................................................Ngaacuguguauggacaucagu....................   1     1   Mm1
......................................................................agaacugugAauggacaucagu....................   2     1   Mm1
......................................................................agaaGuguguauggacaucagu....................   1     1   Mm1
......................................................................agaacuguguauggaGaucagu....................   1     1   Mm1
......................................................................agaacuguguauggacaCcagu....................   16    1   Mm1
......................................................................agaCcuguguauggacaucagu....................   1     1   Mm1
......................................................................agaacugCguauggacaucagu....................   2     1   Mm1
......................................................................agaacuguguauAgacaucagu....................   1     1   Mm1
......................................................................agaacuguguauggacaAcagu....................   14    1   Mm1
......................................................................agaacuguguauggacaucagu....................   2970  0   Mm1
......................................................................agaacuguguauggacauUagu....................   1     1   Mm1
......................................................................agGacuguguauggacaucagu....................   3     1   Mm1
......................................................................agaacuAuguauggacaucagu....................   2     1   Mm1
......................................................................agaacuguguUuggacaucagu....................   1     1   Mm1
......................................................................agaacuguguauUgacaucagu....................   2     1   Mm1
......................................................................agaacCguguauggacaucagu....................   1     1   Mm1
......................................................................agaacuguguauggacaucaguA...................   10    1   Mm1
......................................................................agaacuguguauggacaucaguU...................   30    1   Mm1
.......................................................................gaacuguguauggacaucagu....................   5     0   Mm1

................................ugauuguccaaacgcaau..............................................................   1     0   Mm2
................................ugauuguccaaacgcaauu.............................................................   2     0   Mm2
................................ugauuguccaaacgcaauuc............................................................   20    0   Mm2
................................ugauuguccaaacgcaauucu...........................................................   81    0   Mm2
................................ugauuguccaaacgcaaCucu...........................................................   2     1   Mm2
................................ugauuguccaaacgcaauucuu..........................................................   3     0   Mm2
................................ugauuguccaGacgcaauucuug.........................................................   1     1   Mm2
................................ugauuguccaaacgcaauucuug.........................................................   11    0   Mm2
................................ugauuguccaaacgcaauuGuug.........................................................   1     1   Mm2
................................ugauuguccaaacgcaauucuugu........................................................   2     0   Mm2
.......................................................uuuuauuguuauucgagaacuguguauggacauU.......................   1     1   Mm2
...........................................................auuguuauucgagaacuguguauggacaucagu....................   1     0   Mm2
..............................................................guuauucgagaacuguguauggacaucag.....................   1     0   Mm2
.................................................................auucgagaacuguguauggacauca......................   2     0   Mm2
.................................................................auucgagaacuguguauggacaucag.....................   1     0   Mm2
.................................................................auucgagaacuguguauggacaucagu....................   1     0   Mm2
..................................................................uucgagaacuguguauggacaucag.....................   2     0   Mm2
.....................................................................Cagaacuguguauggacauc.......................   1     1   Mm2
.....................................................................Cagaacuguguauggacaucag.....................   1     1   Mm2
......................................................................agaacuguguauggacau........................   2     0   Mm2
......................................................................agaacuguguauggacGuc.......................   1     1   Mm2
......................................................................agaacuguguauggacaAc.......................   1     1   Mm2
......................................................................agaacuguguauggacauU.......................   4     1   Mm2
......................................................................agaacuguguauggacauc.......................   51    0   Mm2
......................................................................agaacuguguauggacaucU......................   3     1   Mm2
......................................................................agaacuguguauggacaGca......................   1     1   Mm2
......................................................................agaacuguguauggacauca......................   188   0   Mm2
```

Star Mature

```
gcaucuuccuucguaagaaagguuacaggcggugauuguccaaacgcaauucuuguuuuauuguuauucgagaacuguguauggacaucagugcuugccggccgguuucgac
......................................................................agaacuguguauggacauUa......................  1     1  Mm2
......................................................................Cgaacuguguauggacauca......................  1     1  Mm2
......................................................................agaacuguguUuggacaucag.....................  2     1  Mm2
......................................................................agaacuguguauggacaucGg.....................  2     1  Mm2
......................................................................agaacuguguauggacaucag.....................  896   0  Mm2
......................................................................agaacuguguauggacUucag.....................  4     1  Mm2
......................................................................agaacuguguauggacaAcag.....................  2     1  Mm2
......................................................................agaacuguguGuggacaucag.....................  1     1  Mm2
......................................................................agaacuguguauggacGucag.....................  1     1  Mm2
......................................................................agaacuguguauggacCucag.....................  1     1  Mm2
......................................................................agaacNguguauggacaucag.....................  1     1  Mm2
......................................................................agaacuguguauggacaCcag.....................  5     1  Mm2
......................................................................agaacuguguauggacaucaU.....................  3     1  Mm2
......................................................................agaacuguguauggacaGcag.....................  4     1  Mm2
......................................................................agaacuguAuauggacaucagu....................  2     1  Mm2
......................................................................agaUcuguguauggacaucagu....................  1     1  Mm2
......................................................................agaacuguguauggacaAcagu....................  12    1  Mm2
......................................................................agaacuguguauggacGucagu....................  2     1  Mm2
......................................................................agaacuguguauggacCucagu....................  3     1  Mm2
......................................................................agaacuguguauggacaGcagu....................  6     1  Mm2
......................................................................agaacuguguauggacaucagA....................  5     1  Mm2
......................................................................agaacuguguauggacauAagu....................  1     1  Mm2
......................................................................agaacuNuguauggacaucagu....................  1     1  Mm2
......................................................................agaacuCuguauggacaucagu....................  1     1  Mm2
......................................................................agaacuguguCuggacaucagu....................  2     1  Mm2
......................................................................agaacuguguauggacaCcagu....................  10    1  Mm2
......................................................................agGacuguguauggacaucagu....................  1     1  Mm2
......................................................................agaacuguUuauggacaucagu....................  2     1  Mm2
......................................................................agaacuguguauggacaucagC....................  2     1  Mm2
......................................................................agaacuguguauUgacaucagu....................  1     1  Mm2
......................................................................agaacuguguauggacaucagu....................  2175  0  Mm2
......................................................................agaacuguguauggacaucaUu....................  1     1  Mm2
......................................................................agaacuguguGuggacaucagu....................  4     1  Mm2
......................................................................agaacuguguauggacUucagu....................  2     1  Mm2
......................................................................agaacuguguUuggacaucagu....................  1     1  Mm2
......................................................................agaacuAuguauggacaucagu....................  1     1  Mm2
......................................................................agaacuguguaCggacaucagu....................  2     1  Mm2
......................................................................agaacugCguauggacaucagu....................  1     1  Mm2
......................................................................agaacuguguauggacaucagG....................  5     1  Mm2
......................................................................Ugaacuguguauggacaucagu....................  1     1  Mm2
......................................................................agaacuguguauggacauUagu....................  3     1  Mm2
......................................................................agaacuguguauggacaucaguA...................  4     1  Mm2
......................................................................agaacuguguauggacaucaguU...................  13    1  Mm2
.......................................................................gaacuguguauggacaucagu....................  5     0  Mm2
.........................................................................acuguguauggacaucag.....................  1     0  Mm2
.........................................................................acuguguauggacaucagu....................  3     0  Mm2
..........................................................................cuguguauggacaucagu....................  2     0  Mm2
```
